# Differential effects of male nutrient balance on pre- and post-copulatory traits, and consequences for female reproduction in *Drosophila melanogaster*

**DOI:** 10.1038/srep27673

**Published:** 2016-06-08

**Authors:** Juliano Morimoto, Stuart Wigby

**Affiliations:** 1 Department of Zoology, Edward Grey Institute, University of Oxford, South Parks Road, Oxford OX1 3PS, United Kingdom; 2 Charles Perkins Centre, University of Sydney, Camperdown NSW 2050, Australia

## Abstract

Male fitness depends on the expression of costly traits involved in obtaining mates (pre-copulatory) and fertilization (post-copulatory). However, very little is known about the nutrient requirements for these traits and whether males compromise their diet to maximize one trait at the expense of another. Here we used Nutritional Geometry to investigate macronutrient requirements for pre- and post-copulatory traits in *Drosophila*, when males were the first or second to mate with females. We found no significant effects of male diet on sperm competitiveness. However, although males self-regulate their macronutrient intake at a protein-to-carbohydrate ratio (“P:C ratio”) of 1:1.5, this ratio does not coincide with their optima for several key reproductive traits: both the short-term (~24 hr) rate of offspring production after a female’s first mating, as well as the total offspring number sired when males were second to mate were maximized at a P:C ratio of 1:9, whereas male attractiveness (latency to mate), were maximised at a P:C ratio of 1:1. These results suggest a compromised optimum diet, and no single diet that simultaneously maximizes all male reproductive traits. The protein intake of first males also negatively affected female offspring production following remating, suggesting a long-term intersexual effect of male nutrition.

In most species, females are polyandrous (females copulate with multiple males) and, as a result, males are likely to encounter both competition over access to mates (pre-copulation) and fertilization (post-copulation)^1^,^2^. These two competitive episodes strongly influence male fitness^3^,^4^. However, the expression of male sexual traits involved in both pre- and post-copulatory competition is thought to be costly, meaning that male fitness is expected to depend on resource availability, including nutrients^5^,^6^.

Macronutrients (i.e. protein, carbohydrates and fats) are vital in the molecular homeostasis of the organism and radically shape the physiology, behaviour and the expression of life-history traits^7^,^8^,^9^,^10^. In insects, the intake of specific macronutrients can affect both lifespan and reproduction in a sex-specific fashion (e.g.^11^,^12^,^13^,^14^). For instance, in both *Drosophila melanogaster*^11^,^15^ and *Telleogrylus commodus*^12^ female lifespan increases under high-carbohydrate and low-protein diets whereas maximal female reproduction generally requires high-protein diets. On the other hand, male lifespan and reproduction can align: in addition to longer lifespan, high carbohydrate intake also increases male sexual performance in the cockroach *Nauphoeta cinerea*^16^,^17^, in the broad-horned beetle *Gnatocerus cornutus*^18^ and in *D. melanogaster*^15^. However, male nutrition has to integrate the needs of both pre- and post-copulatory episodes, and imbalanced diets can impose constraints on male allocation to sexual traits^19^. Although recent studies have attempted to investigate dietary effects on the interrelation between pre- and post-copulatory traits (e.g.^19^,^20^,^21^), few have attempted to characterise the specific macronutrient requirements that maximize male expression of these traits^12^,^15^,^16^,^17^,^18^,^22^,^23^. Most studies in this field have focused primarily on pre-copulatory traits (e.g.^12^,^17^,^23^), and only recently have researchers attempted to address the effects of macronutrient balance on male post-copulatory traits^15^,^16^,^22^,^24^. For instance, recent work indicates that a protein:carbohydrate ratio of 1:2 maximises sperm number and fertility in the cockroach *Nauphoeta cinerea*^23^. Although sperm number can be important for male sperm competitiveness, it does not necessarily predict the outcome of post-copulatory competition (see^25^). Positive linear relationships between carbohydrate intake and fitness have been demonstrated in male fruitflies *D. melanogaster*^15^,^22^. Yet, it is not clear whether carbohydrate is beneficial to males in pre- or post-copulatory sexual selection, or both. This raises important questions: what are the specific nutritional requirements for competitive male pre- and post-copulatory traits? Can male diet maximize pre- and post-copulatory traits simultaneously or do different traits require different diets, meaning that males have to compromise in their dietary choices?

To address these questions, we used the geometric framework of nutrition (Nutritional Geometry^26^) to experimentally investigate the nutritional requirements across male *Drosophila melanogaster* sexual selection episodes. Nutritional geometry is a state-space model to investigate the effects of multiple nutrients on fitness (reviewed in^5^). We had two main motivations for this study: i) to investigate the specific nutritional requirement for male success in both pre- and post-copulatory sexual selection and ii) to determine the nutritional balance sought by males when given a choice of diets. We first depleted male ejaculate reserves (e.g. accessory gland products) by mating males 3 times in succession (see^27^,^28^,^29^,^30^) - which we expected to place males in a condition in which macronutrient acquisition is necessary to replenish their ejaculate reserves. We gave a subset of these males a choice between complementary diets with different concentrations of macronutrient and measured the protein-to-carbohydrate ratio (P:C ratio) males aimed to achieve by self-regulating their diet intake – the “target ratio”. The remaining males were allocated to one of 15 defined diets (“no choice”), which varied in both the P:C ratio and in the concentration of macronutrients^5^. Males in these no-choice diet treatments then assumed either one of two roles: 1) the first male to mate with a virgin female, in which the female was subsequently mated with a competitor male (P1 experiment) or 2) the second male to mate with a non-virgin female who had previously mated with a competitor male (P2 experiment). This design allowed us to scrutinise male whether male dietary self-regulation matches the macronutrient requirements of both pre- and post-copulatory episodes of sexual selection.

## Methods

### Fly stocks and culture

We used a wild-type *D. melanogaster* stock collected from Dahomey (now Benin) in 1970, and maintained in large outbred populations with overlapping generations^31^. We also used a recessive *sparkling*^*poliert*^ mutation (*spa*) strain, which produces a rough-looking eye phenotype when homozygous^32^. The *spa* strain was back-crossed into Dahomey for >5 generations to ensure a standard genetic background^33^. Using wild-type focal males, and homozygous *spa* females and competitor males, allowed us to assign paternity through the progeny’s eye phenotype^33^. All fly stocks were maintained, and all experiments conducted, at 25 °C on a 12:12 light:dark cycle in a non-humidified room (i.e. natural humidity). We used standard sugar-yeast-maize-molasses medium with *ad libitum* yeast to feed all *spa* females and *spa* male competitors (see Supplemental Information for standard fly food recipe). We controlled larval density to avoid effects on adult phenotypes^34^: all experimental flies were raised at a density of ~200 eggs in 75 ml bottles with ~45 ml of standard maize-molasses fly food (see Supplementary Information). Virgin flies were collected on ice anaesthesia within 8 hours of eclosion and kept in single-sex vials of 15–20 individuals for 3 days with *ad libitum* yeast prior to experiments.

### Depletion of male ejaculate reserves

**Figure 1:**
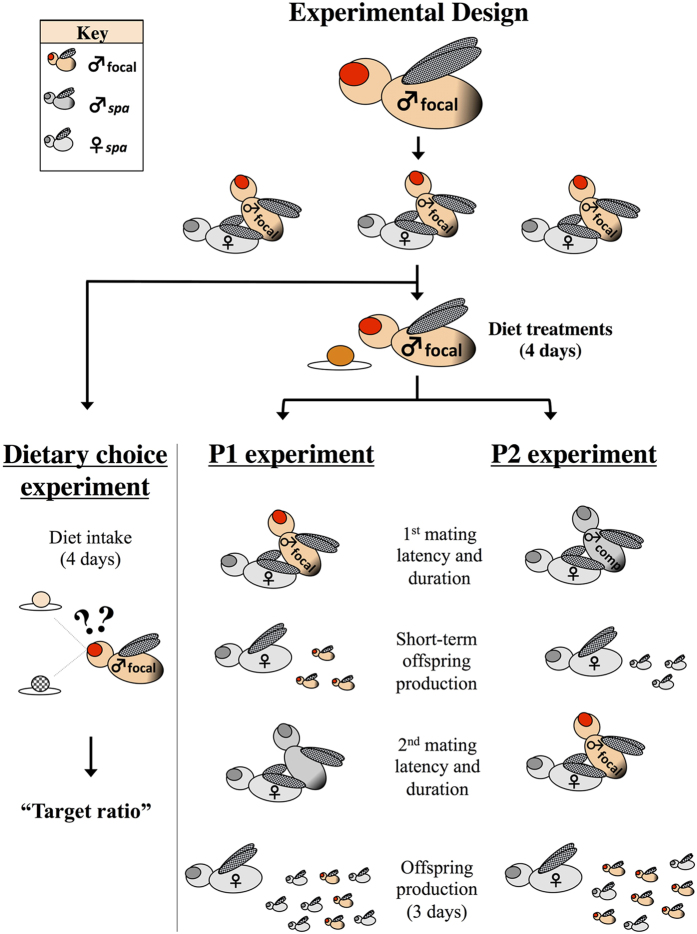
Schematic overview of the experimental design. Virgin wild-type males were kept 3d in standard food with ad libitum yeast for maturation, and then mated with three virgin females consecutively to deplete their ejaculate reserves (i.e. “focal males”). Focal males were then assigned to either a Choice experiment or to 15 distinct defined diets for the P1 and P2 experiments (see Methods for details).

The first steps of all experiments were identical and therefore are explained together until the point in which the experimental procedures diverge (Fig. 1). We allowed virgin wild-type males to mature in standard maize-molasses fly food with *ad libitum* yeast for 3d after emerging from pupae in same sex groups of 15 individuals. We then transferred focal males to fresh vials also with standard food and *ad libitum* yeast and conduced 3 matings to deplete male ejaculate reserves, as follows. We placed each male with one wild-type virgin female and allowed him to mate. After the first mating, we substituted the female for a fresh wild-type virgin female. This process was repeated until our focal males had mated exactly 3 times. Males were given up to 10 h to complete the 3 matings. Three consecutive matings can dramatically reduce the amount of seminal fluid transferred to females due to ejaculate depletion^27^. Therefore our treatment was likely place males in a condition in which nutrient acquisition is necessary to replenish male’s ejaculate reserves, and possibly also energy reserves for courtship. Males that successfully mated with 3 females (i.e. henceforth “focal males”) at the end of 10 h were randomly allocated to one of the three experiments (see below). Males that failed to mate with 3 females were discarded. For the P1 and P2 experiments, focal males were randomly allocated to one of the 15 diet treatments described above and maintained on these diets for 4 days before the experimental matings.

### Diet preparation

Focal males were maintained in vials containing agar/water/nipagin medium sealed with Parafilm into which a 5 μL capillary with liquid diet was placed (adapted from the CAFE assay^11^,^35^). To prepare the diets, we used sucrose as source of carbohydrate (MP Biomedicals, cat. 194018) and hydrolysed yeast as source of protein (cat. 103304). We used sterilized distilled water, filtered in micro-filter of Merck Millipore with pores of 0.22 μm in a sterilized flow, and replaced from the capillaries every ~18 h to minimize bacteria contamination. Diet intake was measured using a digital calliper.

### Experimental design

We performed 3 experiments: (1) a *dietary choice experiment* - where focal males, held singly, were given a choice between complementary diets, and dietary choice was assessed. This experiment allowed us to investigate the P:C ratio males choose when given the opportunity to balance their diet; (2) *P1 experiment* - where focal males were the first to mate with a virgin *spa* female, and females were subsequently given the opportunity to remate with a competitor *spa* male; (3) *P2 experiment* - where *spa* females were first mated to a *spa* male and subsequently given the opportunity to mate with a focal male (Fig. 1)^5^.

#### Dietary choice experiment

Focal males were given a choice between two capillaries with diets with P:C ratios of 1:16 and 3:1, both diets at one of the three concentrations (45 g/L, 90 g/L and 180 g/L). The cumulative intake of both diets were measured every ~18 h for 4d, after which focal males were discarded. The experiment was conducted in two replicates (total N = 68 males). The P:C ratio that males aimed to reach is referred to as ‘the target ratio’ (see^5^ for a review of the method).

#### P1 experiment

We used P:C ratios of: 1:16, 1:9, 1:3, 1:1 and 3:1, each with 3 different concentrations (45 g/L, 90 g/L and 180 g/L) for a total of 15 diets prepared as described above (see “Dietary choice Experiment”). The average intake of protein and carbohydrate in all diets is given in Table S1. After 4 days on one of the 15 the diet treatments, focal males were transferred to fresh vials containing one virgin *spa* female on standard maize-molasses food with *ad libitum* yeast. Flies were allowed to interact until mating was observed. Immediately after a single mating, or if no mating occurred in 4 hours, focal males were discarded. The latency of females to mate with the focal male – a measure of male pre-copulatory attractiveness^36^ and copulation duration were recorded. Mated females were retained and allowed to lay eggs until the following day. 24 hrs after their initial mating the *spa* females were moved to fresh vials with *ad libitum* yeast granules, and 1 *spa* male (competitor male) was added to each vial. The pair were allowed to interact for up to 8 hours or until a single copulation was observed. *spa* males, and females that failed to remate, were discarded. Females that mated with *spa* males (89 out of the initial 210) were immediately transferred into fresh vials with standard maize-molasses and yeast *ad libitum* for egg-laying every 24 h for 3 days, after which females were then discarded. Females that did not remate to *spa* males were discarded. The offspring produced from eggs laid in vials in which females spent the period between matings were used as an estimate of short-term offspring production (including offspring produced by females that did not remate), which is an important component of both male and female reproductive success, since both sexes potentially benefit from a high rate of offspring production soon after the first mating^37^. We also measured the total offspring produced by females that successfully mated with both the 1^st^ and 2^nd^ males, as well the total offspring sired by the focal male (i.e. the wild-type-eyed offspring) over the total of 4 days (i.e. the “short term” offspring, plus those offspring produced after the female remated). We calculated P1, the proportion of offspring sired by the focal males relative to all offspring produced by females after their second mating (see^24^) as an measure of focal male success in sperm competition. All adult offspring were counted 15–17 days after oviposition to allow ample time for development and eclosion at 25 °C.

#### P2 experiment

The design of the P2 experiment was conduced identically to the P1 experiment (see above), except that the *spa* competitor male was the 1^st^ to mate with the female and the focal male was the 2^nd^ to mate with the female. 105 out of 221 females mated with both the *spa* and the focal male. We counted the number of wild-type eyed offspring (sired by the focal male) and *spa* offspring (sired by the *spa* male) produced by females that copulated with focal males, and calculated P2 - the proportion of offspring sired by focal males as the second male to mate - as a measure of sperm competitiveness. For all experiments, the evaporation rate was measured as the average loss of diet in three empty vials (i.e. no flies) *per* diet *per* concentration.

### Data analysis

We first standardised the intake measure of both macronutrients as follows:


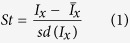


where I_x_ is the individual intake of the macronutrient (protein or carbohydrate), 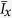 is the mean male intake of that macronutrient and *sd*(*I*_*x*_) is the standard deviation of the male intake of that macronutrient^16^. For the P1 experiment we tested latency of females to mate with focal males, duration of mating with the focal males, the short-term offspring production (between the first and second matings), the proportion of females that remated, P1 paternity share, total offspring sired by the focal male, and total offspring production of the females. We analysed the same factors for the P2 experiment except without the short-term offspring production, and P2 paternity share instead of P1.

We used a general linear model for the analysis of duration of the focal male matings. We used a generalized model (GLM) with quasipoisson errors for the analysis of count data such as the short-term offspring production, total offspring sired by the focal male, total offspring production of female (P1 experiment), and offspring production of females after the 2^nd^ mating (P2 experiment). For proportion data (i.e. P1 and P2 paternity share, the proportion of females that remated), we used a GLM with quasibinomial errors. We fitted a GLM with a gamma error to evaluate the effects of latency of females to mate with focal males; latency was square-root transformed to improve model fit in the P2 experiment. For the P1 latency analysis we included all females (including those that subsequently failed to remate). We Box-Cox transformed the mating duration data for the focal male mating in the P1 experiment to best approximate normality of residuals. In addition to the main effects of P and C intake, we controlled for confounding variables by adding the following covariates in models where appropriate: intermating period (i.e. period between the 1^st^ and 2^nd^ mating), duration of 1^st^ and 2^nd^ mating, latency to 1^st^ mating, short-term productivity, and total female productivity (see Supplemental Tables for details). We provide the output of the analyses in Tables S2–S7 together with the raw data of the reproductive traits measured in this study (Figure S1 and S2) in the Supplementary Information. To visualise the nutritional landscapes, we used the functions ‘Tps’ and ‘surface’ of the ‘fields’ package in R, which is a standard method for visualization of nutritional geometry data (e.g.^5^,^11^,^15^). All nutritional landscapes are of raw (non-transformed) data. For the dietary choice experiment, we measured male total volume intake and total macronutrient intake using ANOVA with diet concentration (i.e. 45 g/L, 90 g/L and 180 g/L) controlling for experimental replicate, followed by a post-hoc Student-Newman-Keuls test (α = 0.05) using the ‘agricolae’ package in R. We confirmed that the ANOVA residuals approximated normality. We used paired *t-test* to compare the observed and the predicted intake of carbohydrates. The predicted carbohydrate intake was calculated as the total individual macronutrient intake – i.e. sum of the intakes of protein and carbohydrates - multiplied by the P:C ratio of interest (e.g. predicted carbohydrate intake in a P:C ratio of 1:1 = total male macronutrient intake * 0.5). All analyses were performed using R version 3.2.2^38^.

## Results

### Dietary choice experiment

**Figure 2:**
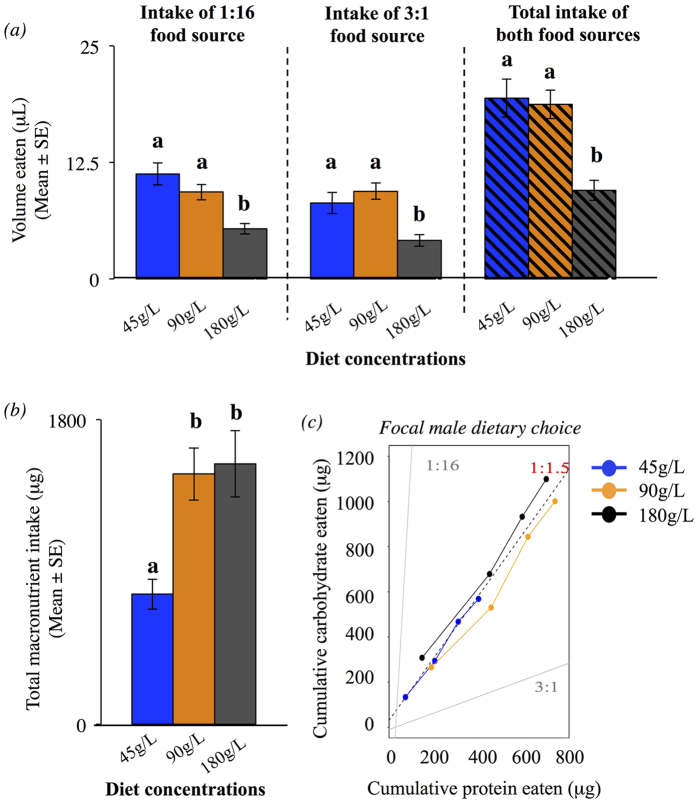
Male dietary choice experiment. (**a**) *Solid bars:* Male volume intake (in μL) of the 1:16 and 3:1 food sources across the three experimental concentrations (i.e. 45 g/L, 90 g/L and 180 g/L). *Striped bards:* The sum of the intake of both food sources (“total intake of both food sources”). Note that male volume intake is higher in 45 g/L and 90 g/L diets (post-hoc SNK-test (α = 0.05)). Blue bars – diet concentration of 45 g/L; Orange bars – diet concentration of 90 g/L; Dark grey bars – diet concentration of 180 g/L. (**b**) Total male macronutrient intake (in μg) across the three different concentrations (i.e. 45 g/L, 90 g/L and 180 g/L) in the dietary choice experiment. Post-hoc SNK-test (α = 0.05). Blue bar – diet concentration of 45 g/L; Orange bar – diet concentration of 90 g/L; Dark grey bar – diet concentration of 180 g/L. (**c**) Cumulative intake of protein and carbohydrate (in μg) and the target ratio of 1:1.5 of male in our experiment. Dark-grey solid lines represent the two dietary choices available for males (i.e. 1:16 and 3:1 P:C ratios). Black dashed line – Target ratio of 1:1.5. Blue line – diet concentration of 45 g/L, Orange line – diet concentration of 90 g/L and Black line – diet concentration of 180 g/L.

We first investigated the P:C ratio regulated by males when given the opportunity to balance their macronutrient intake. Despite having a significantly higher volume intake in the 45 g/L diets (*1:16 food source*: ANOVA_Concentration_: F_2,64_ = 11.459, p < 0.001; *3:1 food source:* ANOVA_Concentration_: F_2,64_ = 9.240, p < 0.001; *Total volume intake:* ANOVA_Concentration_: F_2,64_ = 12.267, p < 0.001, Fig. 2a), the total macronutrient intake was still lower when males fed on a 45 g/L diet (ANOVA_Concentration_: F_2, 64_ = 10.17, p < 0.001, Fig. 2b). In diet concentrations of 90 g/L and 180 g/L, males reached similar macronutrient intake, but compensated the difference in diet concentration by eating a higher volume of food when fed a 90 g/L diet (Fig. 2a,b); this volume was similar to the volume eaten by males in 45 g/L diets (Fig. 2a). Together, these results suggest that males can compensate the low food concentration by eating more, but there is a point beyond which the food is too diluted for compensation to be attained (i.e. males are not physically or physiologically able to eat more). Importantly, across all concentrations males consumed a P:C ratio ~1:1.5 (45 g/L, P:C = 1:1.57; 90 g/L, P:C = 1:1.3; 180 g/L, P:C = 1:1.58; Mean P:C = ~1:1.5; Fig. 2c) suggesting that males biased their macronutrient intake to high carbohydrates. In total, males ate an average of approximately 590 μg of carbohydrates and 400 μg of protein. Males had a higher carbohydrate intake than expected in a 1:1 ratio (*t*_*67*_ = 6.544, p < 0.001), and a lower carbohydrate intake than expected in a 1:9 ratio (*t*_*67*_ = −12.784, p < 0.001), confirming that male dietary choice tends to have a slightly higher carbohydrate intake than a balanced diet of P:C ratio 1:1. Knowing the P:C ratio males aimed to achieve, we hypothesized that this ratio maximized either pre- or post-copulatory traits, or both. We then fed males on 15 diets with fixed P:C ratios and performed P1 and P2 experiments (see Methods).

### P1 experiment – focal male 1^st^ to mate

#### Dietary effects on pre-copulatory traits

**Figure 3:**
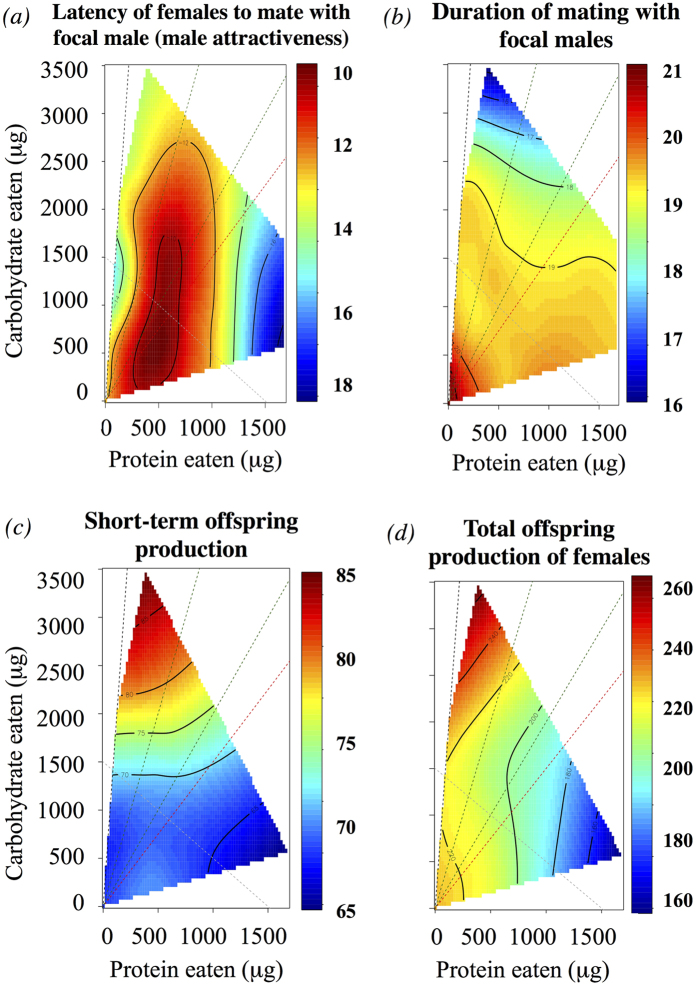
Nutritional landscapes of the P1 experiment, in which male mated with virgin females. (**a**) Female latency to mate with the focal male (min) – i.e. focal male attractiveness. Note that higher male attractiveness (low latency) is indicated by hotter colours. (**b**) Duration of the focal male mating. (**c**) Short-term (~24 h) offspring production after mating with the focal male. (**d**) Total offspring production of females (including offspring of the 1^st^ and 2^nd^ males). For guidance, we included reference lines in the graph: Grey dashed line is the caloric isocline (i.e. line in which the calories are the same); Black dashed line represents the most carbohydrate-rich diet used in our experiment (i.e. P:C ratio of 1:16); Red dashed line stress the P:C ratio of 1:1.5 or the “target ratio” and green dashed lines show the P:C ratio of 1:4 and 1:2.

There was a marginally non-significant quadratic effect of focal male protein intake on our measure of male attractiveness to virgin females (the latency of virgin females to mate with the focal male), in which the nutritional landscape peaked at an intermediate protein intake of ~500 μg and a low-to-intermediate carbohydrate consumption (~150 μg to ~1500 μg; Dispersion: 0.218; Protein*Protein: F_1,206_ = 3.454, p-value = 0.064; Table S2) with P:C ratios lying between 3:1 and 1:3 (Fig. 3a).

#### Dietary effects on mating duration and post-copulatory traits

We found that mating duration was negatively influenced by carbohydrate intake (Dispersion: 62.455; Carbohydrate: F_1,102_ = 4.134, p-value = 0.043; Table S3, Fig. 3b). Maximum mating durations corresponded to the low carbohydrate and protein intakes and P:C ratios between 1:4 and 1:9. We found no significant effect of macronutrients on the latency of females to mate with the 2^nd^ male or proportion of females that subsequently remated with the 2^nd^ male (Tables S2 and S4). Increasing carbohydrate intake had a significant effect on short-term offspring production (offspring produced before remating; Dispersion: 6.38; Carbohydrate: F_1,86_ = 4.293, p-value = 0.041; Table S5), and the nutritional landscape peaked at a high carbohydrate intake (~3500 μg) and a P:C ratio of ~1:9 (Fig. 3c). We did not find a significant effect of macronutrient intake on the total offspring sired by males over the entire period of the experiment (Table S5). However, the total number of offspring produced by females (which includes both those sired by the focal and competitor male) was negatively influenced by focal (1^st^ male) dietary protein intake (Dispersion: 14.24; Protein, F_1,87_ = 6.830, p-value = 0.010; Table S5), and there was a marginally non-significant quadratic trend for a carbohydrate effect (Carbohydrate^*^ Carbohydrate: F_1,84_ = 3.595, p-value = 0.061; see Table S5); inspection of the nutritional landscape showed that the total number of offspring produced by females was the greatest at high male carbohydrate intake (~3250 μg) and low male protein intake (~360 μg) also in a P:C ratio of ~1:9 (Fig. 2d). However, neither protein nor carbohydrate significantly influenced P1 (i.e. the proportion of offspring sired by focal males as the first male to mate; Table S6).

### P2 experiment – focal male 2^nd^ to mate

#### Dietary effects on pre-copulatory traits

**Figure 4:**
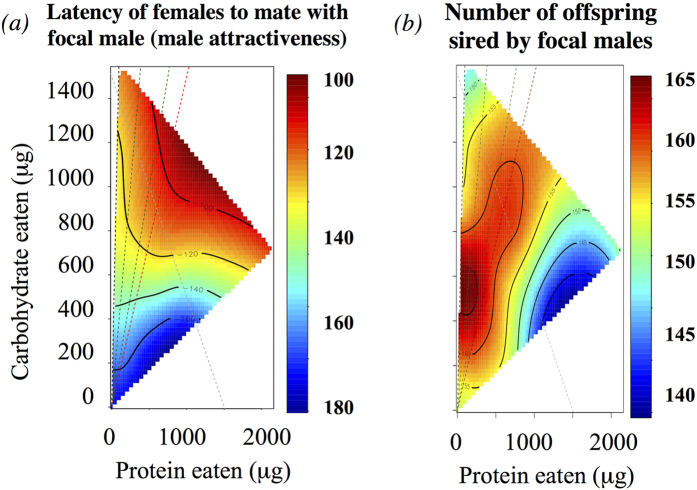
Nutritional landscapes of the P2 experiment, in which males mated with non-virgin females. (**a**) Female latency to mate with the focal male (min) – i.e. focal male attractiveness. Note that higher male attractiveness (low latency) is indicated by hotter colours. (**b**) The number of offspring sired by the focal male. For guidance, we included reference lines in the graph: Grey dashed line is the caloric isocline (i.e. line in which the calories are the same); Black dashed line represents the most carbohydrate-rich diet used in our experiment (i.e. P:C ratio of 1:16); Red dashed line stress the P:C ratio of 1:1.5 or the “target ratio” and green dashed lines show the P:C ratio of 1:4 and 1:2.

We found a non-significant trend for increased carbohydrate intake to reduce the latency of females to mate with the focal male (i.e. an increase in male attractiveness; Dispersion: 0.333; Carbohydrate: F_1,104_ = 3.044, p-value = 0.084; Table S2), which measures male attractiveness when focal males encounter an unreceptive females; there was also a borderline non-significant interaction protein*carbohydrate on the latency of females to mate with the focal male (Protein*Carbohydrate: F_1,99_ = 3.799, p-value = 0.054, Table S2). Together, these results showed that the nutritional landscape for male attractiveness as a 2^nd^ male to mate with unreceptive females peaked at relatively high macronutrient intakes (~1100 μg of each of the macronutrients) and a P:C ratio of ~1:1 (Fig. 4a). We found no effect of focal male macronutrient intake on the proportion of females that remated with the focal male (Table S4).

#### Dietary effects on mating duration and post-copulatory traits

Focal male macronutrient intake had no significant effect on the duration of the focal male mating (Table S3). There was a significant interaction between protein and carbohydrate intake on the number of offspring sired by the focal male (Dispersion: 25.03; Protein*Carbohydrate: F_1,97_ = 4.598, p-value = 0.034, Table S7), in which the number of offspring sired by the focal male was the greatest at a low protein intake (~60 μg) but at an intermediate carbohydrate intake (~550 μg) in a P:C ratio of ~1:9 (Fig. 4b). However, there was no significant effect of focal male macronutrient intake on the total number of offspring produced by females (including those sired by both the *spa* 1^st^ male and the focal 2^nd^ male; Table S7), or on the proportion of wild-type offspring sired (P2) by the focal males (Table S6).

## Discussion

Our results reveal that macronutrient intake, particularly of carbohydrates, can influence male pre and post-copulatory reproductive traits. The data suggest that there is a mismatch between male dietary choice and the dietary requirement for specific reproductive traits. When given the opportunity to self-regulate their diet, males seek a P:C ratio of ~1:1.5. However, this ratio is different from the P:C ratios that maximized offspring production (~1.9; Fig. 3c) and male attractiveness (~1.1; Fig. 3a). These results show that males may be compromising their dietary intake to maximize different pre- and post-copulatory traits since there is not a single diet that maximizes all male reproductive traits. We also found that increasing male protein intake significantly reduced the total offspring productivity of their mates, suggesting an intersexual effect of male diet on female offspring production. Below, we discuss the main findings of this study.

### Male dietary choice

The data show that males compensate the macronutrient dilution in the diet by eating more food, as found previously for female *D. melanogaster*^11^. However, there is likely either a physical or physiological constraint (or both) that constrains male maximum food intake. If diets are too diluted, as in the case of our 45 g/L concentration, males may not be able to consume sufficient liquid to reach their optimum macronutrient intake (Fig. 2a,b). Despite this, males in our experiment still balanced their nutrient intake to a ~1:1.5 P:C ratio even when they failed to reach their optimum total macronutrient intake (Fig. 2c). This carbohydrate-rich diet is likely to have fitness benefits but it does not coincide exactly with the peaks of the nutritional landscapes of the reproductive traits measured in this study (see ‘Results’). These results, together with the findings of^15^,^22^ in *D. melanogaster*, provide evidence that males may have to compromise their diet balance intake, because there is not a unique diet capable of maximizing all male traits.

There have been several explanations for why males do not balance their diet to maximize their fitness traits (e.g.^12^,^15^). First, males might maximize a trait that has not been measured in the study^15^ or regulate nutrient intake to maximize multiple, competing traits^16^,^17^. However, males might also be constrained in their dietary balance for the expression of fitness traits because of their shared genome and dietary preferences with females (“sexual conflict over nutrition”), which might shift male choice to a diet balance that is not optimal for male’s fitness (e.g. see discussion in^12^). Sexual conflict over nutrition was proposed as one of the explanation for male non-optimal dietary balance in *D. melanogaster*^15^ (but see^14^), *T. commodus*^12^ and *Gryllodes sigillatus*^23^.

### Dietary effects on the production of offspring

The data show that male carbohydrate intake in a P:C ratio of ~1:9 significantly maximizes both short-term offspring productivity after mating with virgin females, and the number wild-type offspring sired by males after mating with non-virgin females (Figs 3c and 4b). These results are consistent with the broader results of^15^, in which male *D. melanogaster* lifetime offspring siring increases with male carbohydrate intake. The mechanisms underlying the effects of macronutrient on offspring siring are not known, although several potential routes for this effect are possible. For instance, in adult male cockroaches *Nauphoeta cinerea*, carbohydrate intake in a P:C ratio of ~1:2 maximizes male sperm number^16^, showing that adult male carbohydrate can influence ejaculate components. In *D. melanogaster*, sex-peptide (SP), a seminal fluid protein present in the male ejaculate and known for increasing egg laying and reducing female receptivity^39^,^40^,^41^,^42^,^43^, binds to and is gradually released from sperm^40^,^42^,^44^,^45^. Therefore, it is possible that the effects found in our study might be linked directly to SP or sperm, or to an interaction between sperm number and SP transference and release. Other seminal fluid proteins could also be affected by male macronutrient intake. For instance, *D. melanogaster* ovulin is a peptide present in the male ejaculate that controls female egg laying in the first day after mating^46^,^47^, and a suite of other seminal proteins are required for long-term female post-mating responses^48^,^49^. If the expression of any of these proteins is under dietary regulation then diet will inevitably have consequences for male reproductive success.

We also found that high protein intake has a significantly strong negative effect on the total offspring productivity of their mates in the P1, but not P2, experiment. Although there are several mechanisms that can underlie intersexual effects of macronutrient intake, a change in ejaculate traits is again a likely candidate^50^. For instance, if male macronutrient intake alters the production or transference of SP, ovulin, or other proteins required for full female post-mating responses, this could ultimately affect female productivity (see discussion above). In addition, a surplus of protein intake has been hypothesised to be toxic for both males and females, even though it maximises female egg production^11^,^12^,^13^,^15^,^51^. Thus, high protein intake might impair male ejaculate production or reduce ejaculate quality, which in turn could reduce offspring production of females. These results also suggest an intersexual effect of male nutrition on the offspring production of females. Future studies should address whether male macronutrient intake can also influence offspring traits as well as number, and the potential underlying ejaculate mechanisms.

### Dietary effects on sperm competitiveness

We found no evidence that the intake of macronutrient affected male sperm competitive ability, which was measured as the proportion of offspring sired by the focal males (i.e. P1 or P2) (see^24^ for similar approach). This is consistent with the previous findings in adult male *D. melanogaster*, which showed that yeast concentration (i.e. varying from 20% up to 200% of dietary yeast) did not have any effect on either P1 or P2^24^. Nonetheless, diet can affect the proportion of offspring sired by males if changes in the availability of macronutrients occur during development. For instance, showed that larvae *D. melanogaster* reared in low yeast (i.e. 10% of the normal yeast content) developed into smaller adult males that had significantly lower P1^52^. This may suggest a critical window for the development or maturation of male traits involved in sperm competition, in which the lack of essential macronutrients at this critical point affects male adult success in sperm competition, whereas adult dietary changes have a lesser influence on sperm competitiveness.

It should also be noted that in our study both the sperm competition and offspring production measures following female remating were neccesarily taken from females that successfully remated under controlled conditions (i.e. on the day following the initial mating), in both the P1 and P2 experiments. Thus, these females may be particularly susceptible to fast remating. It will be important in future studies to investigate the influences on overall male reproductive success when all females (not just those that remate the following day) are given opportunities to remate multiply, in free-mating conditions and over longer periods, to more closely reflect the conditions under which sexual selection acts in nature (as well as our lab-adapted populations).

### Dietary effects on male pre-copulatory success and mating duration

The results suggest that male dietary requirements to maximize pre-copulatory success are different from the requirements for offspring siring and short-term productivity. We found that male carbohydrate intake has a significant negative effect on the duration of the 1^st^ mating (with the focal male) in the P1 experiment, and that mating duration was maximized at low macronutrient intake (Fig. 3b). In addition, there was a marginal non-significant trend for the interaction of carbohydrate and protein to affect male pre-copulatory attractiveness to previously mated, unreceptive females, whereby males performed better under a high macronutrient with a P:C ratio of ~1:1 (Fig. 4a). Encountering unreceptive females is likely common to males *D. melanogaster* as females in this species are polyandrous^53^. Therefore, male attractiveness to unreceptive females is expected to play a significant role in determining male fitness. This is consistent with findings for the cockroach, *Nauphoeta cinerea*, for which a positive effect of carbohydrate intake on pheromone production and male attractiveness has been reported^17^. In males *D. melanogaster*, sexual attractiveness and female recognition are influenced by volatile pheromones and cuticular hydrocarbons^54^,^55^,^56^, and future studies will reveal whether male carbohydrate intake increases male sexual attractiveness by modulating the production of these compounds. Carbohydrate intake might also contribute to male energetic supply during courtship. For instance, male carbohydrate intake is positively associated with male calling effort in *Teleogryllus commodus*, which is a trait used by males to attract females, suggesting that dietary carbohydrate may be used as a direct source of energy for courtship^12^.It is important to note, however, that we only detected a marginally non-significant trend in this study, that the macronutrient effects on mating latency in the P2 experiment are for females that were willing to remate in the experiment (as with the sperm competition measures – see discussion above), and that we detected no significant effects of macronutrients on the proportion of females that remated (Table S4). Thus, the overall importance to male reproductive success of macronutrient intake effects on mating latency with previously mated females requires further investigation. We also found a non-significant trend for intermediate intake of protein (~500 μg) to increase male attractiveness to receptive (virgin) females in P:C ratios from 1:3 and 3:1. This result is consistent with the idea that male diet can influence pre-copulatory attractiveness to both virgin and previously mated females, and the data from both experiments are consistent with the idea that macronutrient intake has different effects on male pre- and post-copulatory traits.

It is important to note that the data in our experiment was taken from males under a specific set of conditions. Males were 8 days old at the time of measurements, and had been experimentally depleted of ejaculate prior to treatments. The effect of previous mating history on dietary effects and dietary choices remains to be tested, although there is currently no evidence that age alters the P:C ratio chosen by male *D. melanogaster* (see^15^). More generally it will be interesting for future studies to test whether flies make dynamic choices throughout their lives based on social experience, and whether there are affects of different types of social interactions (e.g. mating, male-male aggression) Furthermore, it will be important to determine whether the ability to choose diets *per se* influences male performance, by comparing individuals given diet choice *versus* no-choice.

The specific mechanisms that link male diet to the reproductive traits measured in this study remain to be elucidated. Some effects may be part of a more general behavioural and physiological response to diet. For example, non-optimal diets in general should reduce the pool of resources available to males to allocate to specific traits. This could potentially lead to an overall change in male locomotor activity which might explain the effects on copulation duration and the trends in latency to mating. Similarly, changes to post-copulatory traits might map onto a more general physiological response to diet. However, that fact that our results show macronutrient intake having effects on different traits (or no effects on some traits) suggests that the link between macronutrients and male reproductive traits are more complex than simply a non-specific loss of optimal condition under non-optimal diets. Our results support the idea that some traits are much more sensitive to diets than other traits, and that different diets maximise different traits.

## Conclusions

Overall, our results show male dietary compromise, whereby male dietary choice does not coincide with specific male nutritional requirements for traits involved in pre- and post-copulatory sexual selection. Our study also supports previous findings on the strong effects of carbohydrate intake on male reproduction, which seems to be consistent across insect species^12^,^15^,^16^,^17^,^23^. This taxonomic consistency suggests a tentative possibility of a widespread effect of carbohydrate on male reproduction in invertebrates. It will be important to know how male nutrition affects the abundance of seminal fluid proteins in the ejaculate and its implications to male fitness, which in turn will likely affect the intensity and operation of sexual selection and sexual conflict. Most species of vertebrates also undergo pre- and post-copulatory sexual selection^57^,^58^,^59^, and future investigations should seek to reveal whether macronutrients have similar effects in these taxa.

## Additional Information

**How to cite this article**: Morimoto, J. and Wigby, S. Differential effects of male nutrient balance on pre- and post-copulatory traits, and consequences for female reproduction in *Drosophila melanogaster*. *Sci. Rep.* **6**, 27673; doi: 10.1038/srep27673 (2016).

## Supplementary Material

Supplementary Information
