# Supplementary material for: Differential effects of male nutrient balance on pre- and post-copulatory traits, and consequences for female reproduction in Drosophila melanogaster
Source: Sci Rep. 2016 Jun 8;6:27673. doi: 10.1038/srep27673 (PMC4897696; doi:10.1038/srep27673)
Supplement: Supplementary Information [file srep27673-s1.pdf]

Differential effects of male nutrient balance on pre- and post-copulatory traits, and consequences for female reproduction in *Drosophila melanogaster*

Authors: Juliano Morimoto[1, 2, *], Stuart Wigby[1]

Affiliations: [1]Department of Zoology, Edward Grey Institute, University of Oxford, South Parks Road, Oxford OX1 3PS, United Kingdom

[2] Charles Perkins Centre, University of Sydney, Camperdown NSW 2050, Australia

*- To whom correspondence should be addressed:

juliano.morimotoborges@zoo.ox.ac.uk

Table S1 – Mean intake of protein and carbohydrate in each nutritional rail of both P1 and P2 experiments.

		P1 experiment				P2 experiment			
**P:C ratio**	**Concentration**	**Carbohydrate**	**±SE**	**Protein**	**±SE**	**Carbohydrate**	**±SE**	**Protein**	**±SE**
**1:16**	**45g/L**	528.23	55.55	33.01	3.47	378.12	65.05	23.63	4.06
	**90g/L**	656.80	75.97	41.05	4.74	971.18	118.19	60.69	7.38
	**180g/L**	1251.52	157.10	78.22	9.81	1100.21	118.54	68.76	7.40
**1:9**	**45g/L**	333.99	36.04	37.11	4.00	378.51	41.95	42.05	4.66
	**90g/L**	1153.67	176.75	128.18	19.63	507.06	88.65	56.34	9.85
	**180g/L**	2400.57	172.40	266.73	19.15	524.44	139.18	58.27	15.46
**1:3**	**45g/L**	531.77	43.47	177.25	14.49	156.55	30.74	52.18	10.24
	**90g/L**	1533.84	92.46	511.28	30.82	315.64	55.20	105.21	18.40
	**180g/L**	912.42	124.83	304.14	41.61	243.41	83.00	81.13	27.66
**1:1**	**45g/L**	130.19	25.33	130.19	25.33	210.76	28.72	210.76	28.72
	**90g/L**	340.35	42.32	340.35	42.32	425.31	56.55	425.31	56.55
	**180g/L**	899.35	108.84	899.35	108.84	388.51	60.73	388.51	60.73
**3:1**	**45g/L**	167.75	16.12	503.25	48.37	222.79	23.76	668.39	71.30
	**90g/L**	244.73	42.65	734.20	127.97	272.24	22.69	816.72	68.09
	**180g/L**	283.70	33.97	851.12	101.92	530.47	32.60	1591.42	97.81

Table S2 - Complete table of the analyses of the female latency to mate with the focal male (i.e. P1 and P2 experiments) and female latency to mate with *a* spa competitor male (P1 experiment). $- Analysis performed with females that successfully mated with the 2nd male, £- Statistical analysis performed with transformed response variable (response^0.5). Bold – p <0.1. * – p < 0.05. ** – p < 0.01

	Latency analysis											
**Covariate**	**Female latency to mate with the focal male (P1 experiment)£**				**Female latency to remate (with a *spa* competitor male) (P1 experiment)**				**Female latency to mate with the focal male (P2 experiment)$**			
	Estimate	*SE*	*F-value*	*p-value*	*Estimate*	*SE*	*F-value*	*p-value*	*Estimate*	*SE*	*F-value*	*p-value*
(Intercept)	3.6220	0.015	-	-	-0.0050	0.0019	-	-	0.0097	0.0016	-	-
P	0.0220	0.016	0.4257	0.514	-0.0000	0.0008	0.052	0.820	0.0012	0.0011	0.105	0.746
C	-0.0070	0.016	0.617	0.433	-0.0006	0.0007	0.129	0.720	0.0019	0.0009	**3.044**	**0.084**
P*P	-0.0150	0.006	3.454	**0.064**	0.0002	0.0004	0.383	0.537	-0.0007	0.0006	1.742	0.189
C*C	-0.0020	0.007	0.092	0.761	0.0003	0.0003	0.899	0.345	-0.0000	0.0004	0.226	0.635
Duration of the 1st mating	-	-	-	-	0.0000	0.0000	0.249	0.619	-0.0000	0.0000	2.100	0.150
Latency to the 1st mating	-	-	-	-	0.0000	0.0000	0.793	0.375	-0.0000	0.0000	**7.983**	**0.005****
P*C	0.0130	0.014	0.979	0.323	0.0007	0.0010	0.497	0.482	0.0030	0.0017	**3.799**	**0.054**
Dispersion:	*0.218*				*0.503*				*0.333*			

Table S3 - Complete table of the analyses of the duration of the mating of the focal male in both P1 and P2 experiments. Bold – p <0.1. * – p < 0.05. @- Statistical analysis performed with transformed response variable (response^0.15).

	**Duration of the mating of the focal male**							
**Covariate**	**P1 experiment@**				**P2 experiment**			
	Estimate	*SE*	*F-value*	*p-value*	*Estimate*	*SE*	*F-value*	*p-value*
(Intercept)	0.1567	0.0070	-	-	103.922	103.937	-	-
P	-0.008	0.0070	0.432	0.511	1.7258	1.4052	0.649	0.423
C	-0,0002	0.0060	**4.134**	**0.043***	-0.8406	0.9255	0.135	0.713
PxP	0.0030	0.0030	1.066	0.302	-0.2557	0.5440	1.208	0.274
CxC	-0.005	0.0030	2.356	0.126	-0.4226	0.4781	0.439	0.508
Duration of the 1st mating	-	-	-	-	-0.0792	0.0768	0.776	0.380
Intermating period	-	-	-	-	-0.0583	0.0712	0.989	0.322
Latency to the 1st mate	-0.0008	0.0003	**6.070**	**0.014***	-	-	-	-
PxC	0.0006	0.0060	0.008	0.926	-1.1078	1.3724	0.651	0.421
Dispersion:	*62.455*				*20.021*			

Table S4 - Proportion of females that remated with the *spa* male (P1 experiment) or the focal male (P2 experiment). The analyses were performed with a GLM model with a quasibinomial error. * – $p < 0.05$.

	Proportion of females that remated							
**Covariate**	**P1 experiment (remating with *spa* male)**				**P2 experiment (rematings with focal male)**			
	Estimate	*SE*	*F-value*	*p-value*	*Estimate*	*SE*	*F-value*	*p-value*
(Intercept)	0.624	0.529	-	-	0.8900	0.5530	-	-
P	-0.173	0.241	0.008	0.927	-0.0977	0.3235	0.377	0.539
C	0.033	0.226	0.996	0.319	0.3371	0.2829	0.919	0.338
PxP	0.068	0.115	0.049	0.823	-0.0060	0.1662	1.363	0.244
CxC	0.086	0.113	0.254	0.614	-0.1715	0.1284	1.633	0.202
Latency to the 1st mating	-0.008	0011	0.137	0.711	-0.0011	0.0038	0.024	0.875
Short-term offspring production	-0.013	0.006	**3.983**	**0.047***	-0.0141	0.0076	4.046	**0.045***
PxC	-0.316	0.227	2.017	0.157	0.4868	0.4560	1.161	0.282
Dispersion:	*1.038*				*1.030*			

Table S5 – Complete table of the analysis of short-term offspring production, total offspring sired by the focal male and total offspring produced by females in the P1 experiment. The analyses were performed with a GLM model with a quasipoisson error. Bold – p <0.1. * p <0.05. ** – p < 0.01.

	**P1 experiment reproductive analyses**											
**Covariate**	**Short-term offspring production**				**Total offspring sired by the focal male**				**Total offspring production of females**			
	Estimate	*SE*	*F-value*	*p-value*	*Estimate*	*SE*	*F-value*	*p-value*	*Estimate*	*SE*	*F-value*	*p-value*
(Intercept)	-0.162	5.498	-	-	-30.383	21.172	-	-	-1.770	4.643	-	-
P	-0.001	0.071	0.035	0.850	-0.210	0.253	0.0001	0.993	-0.110	0.060	**6.830**	**0.010****
C	0.020	0.052	**4.293**	**0.041***	0.153	0.185	0.007	0.930	-0.022	0.044	1.360	0.246
P*P	-0.015	0.028	0.835	0.363	0.111	0.093	0.012	0.910	-0.005	0.025	1.868	0.175
C*C	0.019	0.205	0.811	0.370	-0.131	0.086	0.326	0.569	0.021	0.017	**3.595**	**0.061**
Duration of the 1st mating	0.019	0.004	0.710	0.401	-0.009	0.019	0.888	0.348	-0.005	0.003	2.330	0.130
Intermating period	0.002	0.003	0.604	0.439	0.022	0.014	**5.446**	**0.022***	0.005	0.003	2.210	0.140
Duration of the 2nd mating	0.001	0.004	0.190	0.663	-0.042	0.024	**5.005**	**0.028***	-0.003	0.003	0.971	0.327
Total offspring production of females	-	-	**-**	**-**	0.006	0.002	**9.090**	**0.003****	-	-	-	-
P*C	-0.035	0.067	0.269	0.604	0.053	0.243	0.047	0.827	-0.019	0.058	0.105	0.745
Dispersion:	*6.38*				*31.31*				*14.24*			

Table S6 - Complete table of the analysis of the proportion of offspring sired by the focal male in both P1 and P2 experiments. The analyses were performed with a GLM model with a quasibinomial error. Bold – p <0.1. * p <0.05. [&]- Analysis performed with all females that successfully mated

	Proportion of offspring sired by the focal male							
**Covariate**	**P1 experiment**				**P2 experiment**			
	Estimate	*SE*	*F-value*	*p-value*	*Estimate*	*SE*	*F-value*	*p-value*
(Intercept)	-0.470	0.270	-	-	0.914	30.77	-	-
P	-0.292	0.328	0.953	0.331	-0.076	0.4257	2.306	0.132
C	0.264	0.239	0.052	0.819	0.3107	0.2784	0.469	0.494
PxP	0.168	0.132	0.146	0.703	-0.119	0.1723	0.018	0.890
CxC	-0.159	0.105	1.081	0.301	-0.073	0.1522	0.081	0.776
Duration of the 1st mating	-0.070	0.024	0.135	0713	0.0185	0.0242	0.169	0.681
Intermating period	0.030	0.0018	**3.958**	**0.050**	-0.000	0.0210	0.045	0.831
Duration of the 2nd mating	-0.052	0.0352	**3.609**	**0.061**	0.0396	0.0289	1.530	0.219
Total offspring production of females	0.0006	0.003	0.041	0.838	0.0004	0.0028	0.0101	0.920
Short-term offspring production	-	-	-	-	-0.0052	0.0092	0.592	0.443
PxC	0.063	0.311	0.040	0.840	0.410	0.454	0.984	0.323
Dispersion:	*39.950*				*37.99*			

Table S7 - Complete table of the analysis of offspring sired by the focal male and offspring production of females after mating with the focal male in the P2 experiment. The analyses were performed with a GLM model with a quasipoisson error. Bold – p <0.1. * p <0.05. ** – p < 0.01. *** p <0.001. $- Analysis performed with females that successfully mated with our focal males.

	**P2 experiment$**							
**Covariate**	**Offspring sired by the focal male**				**Offspring production of females after mating with the focal male**			
	Estimate	*SE*	*F-value*	*p-value*	*Estimate*	*SE*	*F-value*	*p-value*
(Intercept)	3.445	9.231	-	-	6.4600	8.887	-	-
P	0.0045	0.123	0.623	0.431	0.0034	0.117	0.001	0.973
C	0.0885	0.081	0.0005	0.982	0.0598	0.077	0.078	0.779
P*P	-0.0406	0.045	0.044	0.833	-0.0186	0.043	0.177	0.674
C*C	-0.0193	0.044	0.926	0.338	-0.0312	0.041	0.740	0.391
Duration of the 1st mating	0.0178	0.006	**6.649**	**0.011***	-	-	-	-
Intermating period	0.0004	0.006	0.019	0.888	-0.0008	0.006	0.0001	0.992
Duration of the 2nd mating (i.e. remating)	0.0089	0.009	1.446	0.231	0.0042	0.008	0.197	0.657
Short-term offspring production of the competitor male	0.0079	0.002	**14.966**	**<0.001*** **	-	-	-	-
P*C	0.2521	0.114	**4.598**	**0.034***	0.114	0.109	1.006	0.304
Dispersion:	*25.03*				*25.94*			

Standard fly food recipe - Recipe for the standard fly food used in this study.

Ingredient	Quantity
**Agar**	
**Water**	
Maize Flour	3600 g
Yeast Powder	732 g
Soya	432 g
**Molasses Mix**	
Malt	3600 g
Molasses	1050 g
Water	2 L
**Nipagin mix**	
Methyl 4-Hydroxybenzoate	148 g
Ethanol	1300 mL
Water	200 mL
**Acid Mix**	
Propionic acid	1 L
Orthophosphoric acid	64 mL

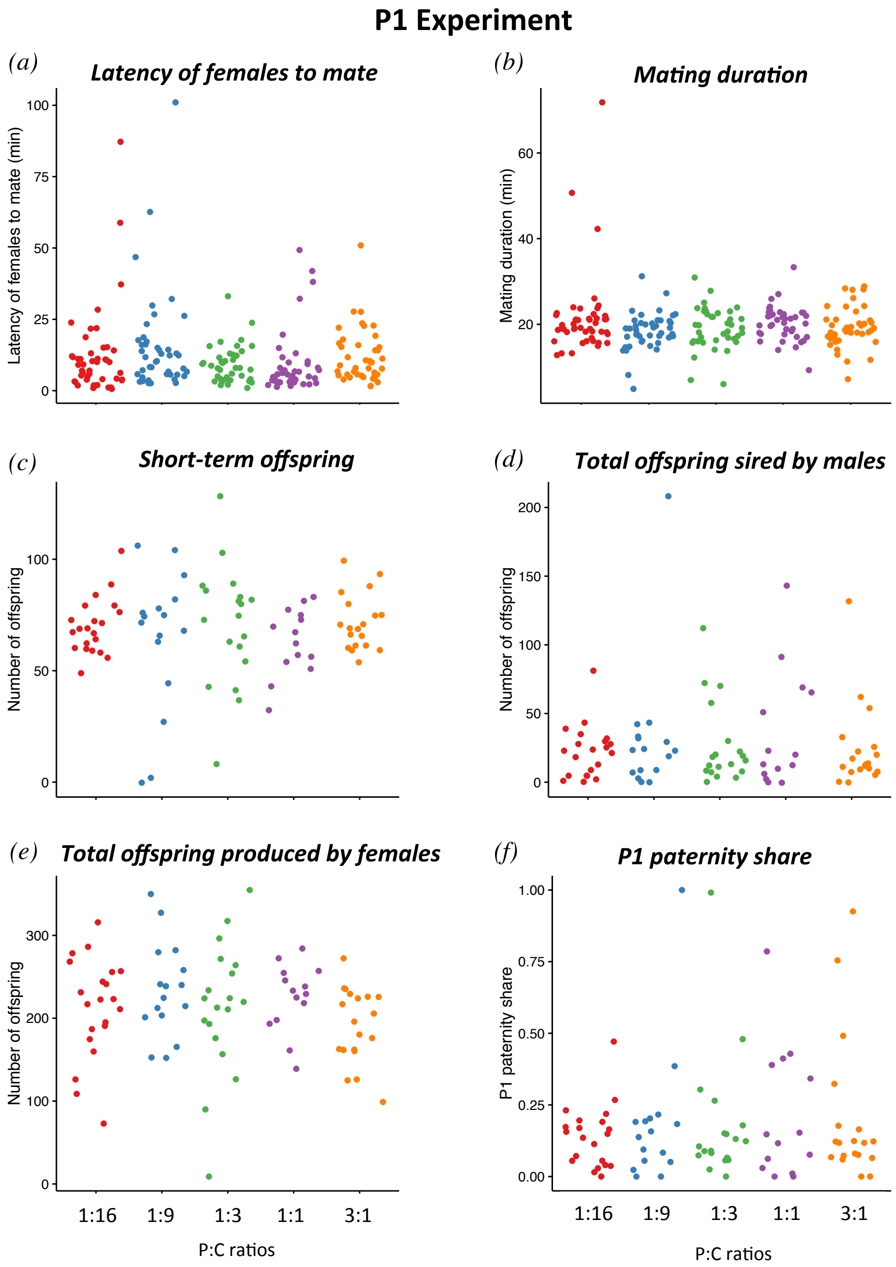

Figure S1 – Raw data of the reproductive traits measured in the P1 experiment. Individual data points are shown. *(a)* Latency of females to mate with the focal male. *(b)* The duration of the focal male mating. *(c)* Short-term offspring production. *(d)* The total offspring sired by males. *(e)* The total offspring produced by females that successfully remated. *(f)* The proportion of offspring sired by focal males with females remated with a competitor male (i.e. P1 experiment).

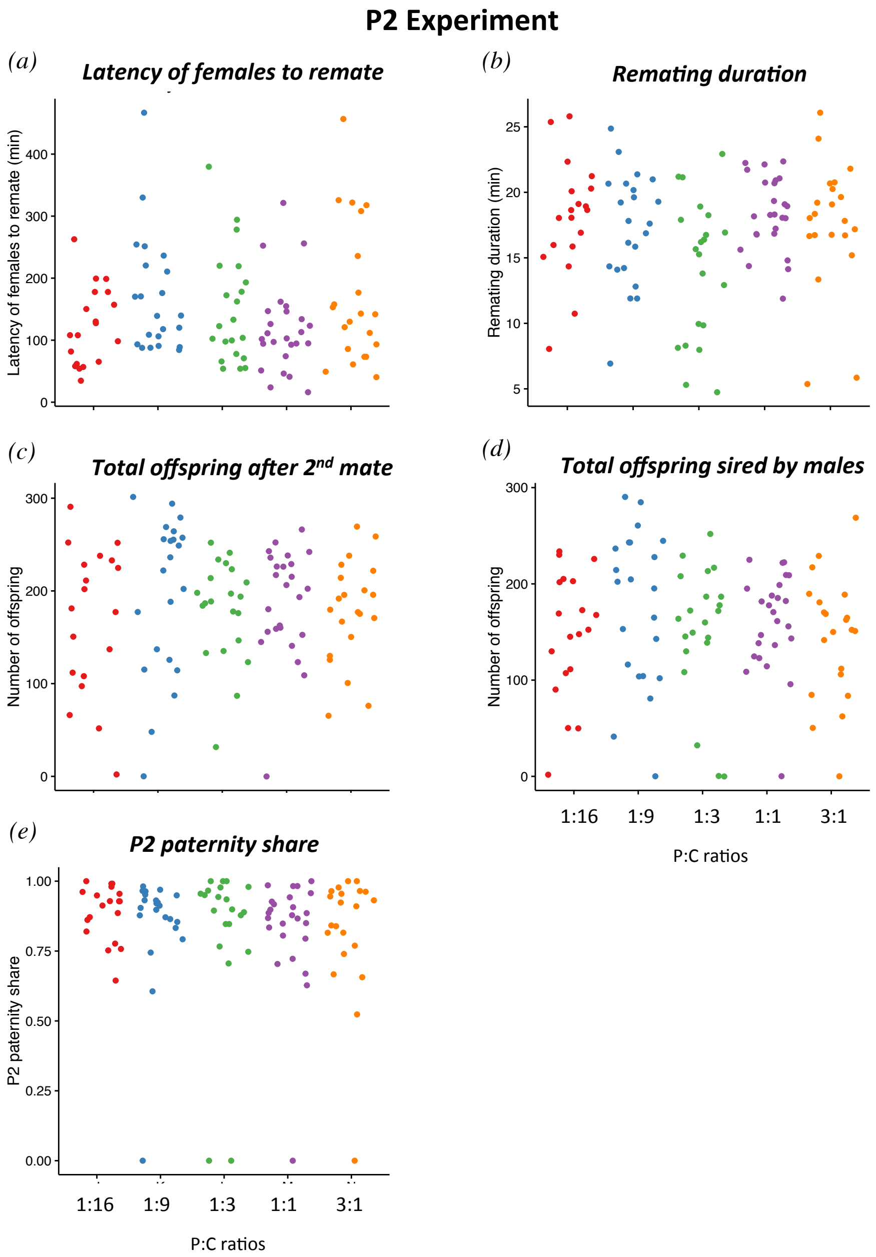

Figure S2 –Raw data of the reproductive traits measured in the P2 experiment. Individual data points are shown. *(a)* Latency of females to remate with the focal male. *(b)* The duration of the focal male mating. *(c)* The total offspring produced by females after remating with focal males. *(d)* The total offspring sired by males. *(e)* The proportion of offspring sired by focal males with unreceptive (previously mated) females (i.e. P2 experiment).
